# The Effect of Carbon Monoxide on the Exergy Behavior of the Lungs

**DOI:** 10.3390/bioengineering5040108

**Published:** 2018-12-07

**Authors:** Juliana Rangel Cenzi, Cyro Albuquerque, Carlos Eduardo Keutenedjian Mady

**Affiliations:** 1 School of Mechanical Engineering, University of Campinas, Campinas 13083-970, Brazil; juliana.cenzi@gmail.com; 2 Department of Mechanical Engineering, Centro Universitário da FEI, São Bernardo do Campo 09850-901, Brazil; cyroan@fei.edu.br

**Keywords:** Exergy analysis, respiratory system, carbon monoxide intoxication

## Abstract

The present work evaluates the impact of carbon monoxide (CO) inhalation on the human lung’s exergy behavior by considering different levels of intoxication and amounts of hemoglobin. Its impact is significant because CO is one of the most common air pollutants in cities and an increase in destroyed exergy may be correlated with lifespan reduction or the malfunctioning of certain human organs. An evaluation of the severity of intoxication as a function of city altitude may intensify the hazard associated with carbon monoxide. A computational model of human lungs obtained from the literature was used to calculate the concentrations of oxygen (O_2_), carbon monoxide (CO), and carbon dioxide (CO_2_) in the respiratory system. With the purpose of better evaluating the different levels of CO intoxication and hemoglobin concentration (which is a function of acclimatization time and some pathologies, such as anemia), a model calculating exergy efficiency for the lungs was proposed. From this model, it was possible to conclude that a higher level of intoxication is associated with lower exergy efficiency values. When associated with carbon monoxide intoxication, higher hemoglobin levels also result in lower efficiency. Eventually, a comparison between previous studies and the current study was carried out, regarding the method employed to calculate the exergy destroyed in the lungs, considering not only gas transport, but also hemoglobin concentration and its reaction with the gases from a second law perspective.

## 1. Introduction

Energy cannot be created or destroyed; it is always conserved, but its ability to produce work can be reduced. This “ability” is defined as another property called exergy, a term introduced by Szargut et al. [1]. It represents the maximum amount of work that can be obtained from a certain type of energy interacting with just the environment. Exergy analysis can be defined as an evaluation of the quality of the energy conversion of a certain process, by evaluating the inefficiency and applying the concepts of entropy generation or destroyed exergy. To properly understand and evaluate these physiological phenomena, a second law analysis may be used as a tool to propose indices. These indices may in turn be applied to thermal comfort [2], sports [3], or health [4].

Zhen [5] states that the second law of thermodynamics is one of the most significant physical laws because it can help us understand life as an irreversible process; it has a beginning, a development, and an end. As for an isolated system, entropy and chronological age can only increase; the production of entropy may be called the “arrow of time.”

Aiming at a better understanding of the exergy behavior of the circulatory system, Albuquerque et al. [6] applied this exergy analysis to the human body to evaluate the relationship between the levels of gases in the lungs, tissues, and the environment with the body’s destroyed exergy. The authors used two control volumes: The lungs and the tissues. It was concluded that most of the destroyed exergy occurs in the tissues. In this article, two hypotheses were used: (1) The reference state of exergy in the human body is the thermodynamic state of the blood O_2_ and CO_2_ in arterial blood; and (2) the blood is treated as a mixture of liquid and ideal gases.

Regarding the analysis of the body as a whole, a method developed by Mady et al. [7,8] was used by Henriques et al. [9]. The latter authors developed and applied an exergy analysis to evaluate human body and respiratory behaviors for different levels of physical activity and environmental conditions. In this paper, the authors evaluated different levels of physical activity, altitudes, and acclimatization periods. As in [6], blood was considered as a mixture of liquid and ideal gases. Nevertheless, the reference state for the exergy analysis was the actual environment where the person was located. The conclusions were that the exergy efficiency for rest conditions decreases as a function of altitude, while the exergy destruction increases for higher altitudes. Acclimatization periods of more than 20 days lead to the highest values of exergy efficiency [9].

Carbon monoxide is produced by the human metabolism, but at too small a rate to cause any damage. Exogenous CO is toxic because its affinity with hemoglobin is 200–300 times greater than with O_2_. Hence, even relatively small concentrations of inhaled CO can reduce delivered oxygen to the tissues, causing hypoxia. The toxicity of CO, therefore, is not caused by any direct effects, but only by the increase of carboxyhemoglobin. Since the reaction between CO and hemoglobin is reversible, the treatment of CO poisoning consists of reducing its inhaled concentration to zero, in order to generate a maximum partial pressure gradient and to increase both alveolar ventilation and O_2_ partial pressure [10].

In this work, exergy analysis was applied to the human respiratory system model proposed by Albuquerque et al. [11] in order to evaluate the degree of intoxication of carbon monoxide and its severity to human health. The exergy model used was proposed by [7,8,9] and adapted to evaluate not only the air, consisting of O_2_, N_2_, and CO_2_, but also CO. Moreover, the blood was not only considered (from the second law perspective) as a mixture of both ideal gases and liquid phases, as in previous studies [9], but hemoglobin and its reaction with O_2_, CO, and CO_2_ was also considered. Simulations were carried out to evaluate different altitudes, exposure times, and CO concentrations.

## 2. Materials and Methods

**Figure 1:**
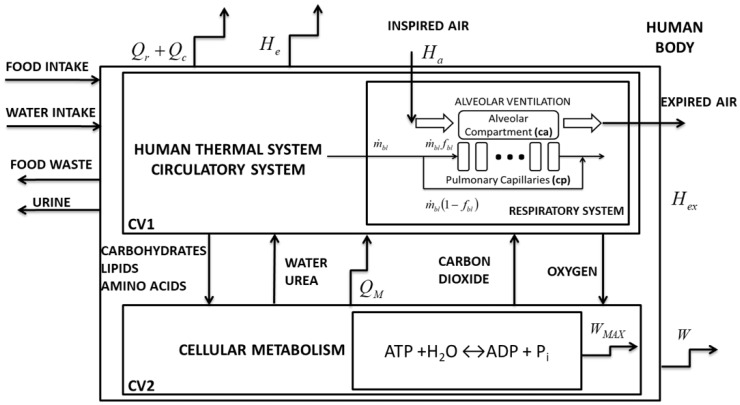
Human body schematic representation with circulatory and respiratory systems, modified from [8]. The respiratory system was obtained from [11].

Mady et al. [8] proposed a model of the human body to apply exergy analysis. This model assumes that the mass flow rate inputs (air, water, food) are equal to the outputs (air, urine, feces) for the period of one day (Figure 1). It has 2 control volumes: The first one represents the thermoregulatory, circulatory, and respiratory systems, and the second one is cellular metabolism. They exchange some flow rates: Nutrients, water, heat, and the byproducts of nutrient oxidation. These exchanges occur with the environment, when combined, exchange work and exergy flow rates associated with vaporization (He), respiration (Hex–Ha), and heat, due to convection and radiation.

**Figure 2:**
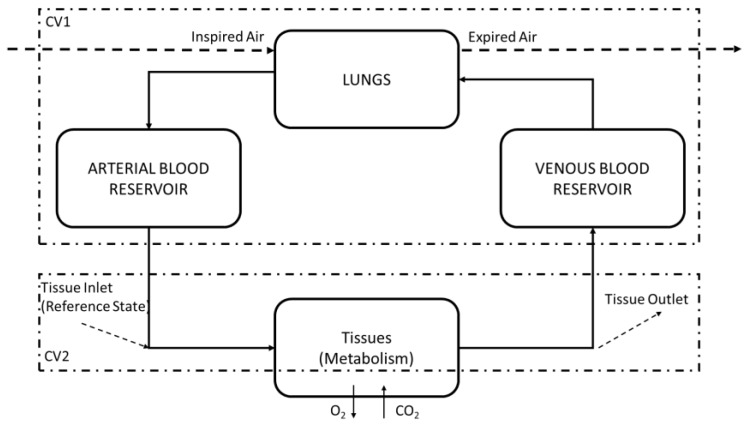
Control volume used by Albuquerque et al., modified from [6].

Albuquerque et al. [6] used the model from Figure 2 to analyze the respiratory system, which has 2 control volumes (CVs). CV1 is used to model the lungs and the arterial and venous compartments, and CV2 represents the tissues (all the other parts of the body and their metabolisms). The tissues represent the places where metabolic reactions and gaseous exchanges take place.

**Figure 3:**
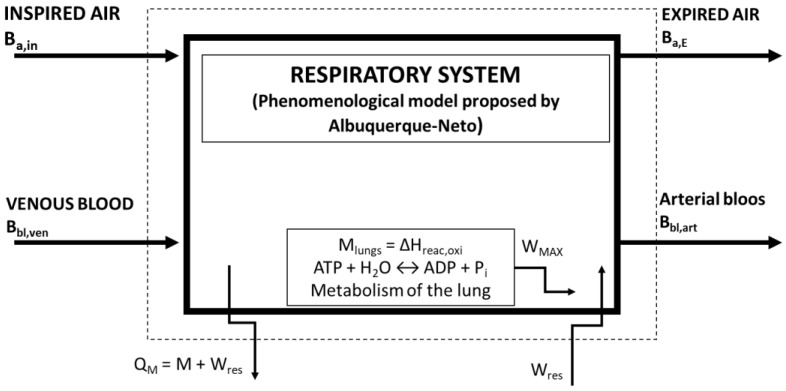
Control volume as proposed based on previous studies of the group [7,8] and used in the present paper. Modified and adapted from [7,8].

Henriques et al. [9], referring to Mady et al. [7], proposed a modification in the control volume. In this new model (Figure 3), the complete respiratory system is found inside the control volume. The main differences here is that the work done in the lungs is considered, the lung metabolism is calculated, and that the air has a more complex composition. This is the control volume used in the present work to apply exergy analysis. The first exergy analysis model discussed in this paper is also based on this work.

To obtain the main streams and energy behavior of the lungs, we used the phenomenological model developed by Albuquerque et al. [11] to evaluate the carbon monoxide concentration in the body compartments, as well as the other gases (O_2_, CO_2_). The exergy destroyed in the lungs is given by Equation (1):

(1) $${\dot{B}}_{d,lung}={\dot{B}}_{M,lung}+{\dot{B}}_{bl,ven}+{\dot{B}}_{a,in}+{\dot{W}}_{resp}-{\dot{B}}_{bl,art}-{\dot{B}}_{a,ex}-{\dot{Q}}_{M,lung}\left(1-\frac{T_{0}}{T_{body}}\right)$$

In this equation, *B_M_*_,*lung*_ is the metabolic exergy of the lungs (exergy variation of the oxidation of nutrients), *B_bl_*_,*ven*_ is the exergy of venous blood, *B_bl_*_,*art*_ is the exergy of arterial blood, and *W_resp_* is the performed power of the intercostal muscles to the lungs. The terms *B_a_*_,*in*_ and *B_a_*_,*ex*_ are the exergy of the air in the inlet and outlet of the lungs, respectively. The term *Q_M_* = (1 − *T*_0_/*T_body_*) is the exergy associated with the metabolic heat transfer, explained in Equation (2). Assuming steady-state conditions, the heat transferred by the lungs is the sum of its metabolism and the work done by the intercostal muscles and the diaphragm to the lungs:

(2) $${\dot{Q}}_{M,lung}={\dot{M}}_{lung}+{\dot{W}}_{resp}$$

Mady and Oliveira Jr. [7] provided empirical correlations that give the values of the energy and exergy metabolism of the lungs. They are proportional to the mass rates of oxygen consumed, carbon dioxide produced, and nitrogen excreted:

(3) $${\dot{M}}_{lung}=11371{\dot{m}}_{O_{2},lung}+2366{\dot{m}}_{CO_{2},lung}-129{\dot{m}}_{N_{2},lung}$$

(4) $${\dot{B}}_{M,lung}=9558{\dot{m}}_{O_{2},lung}+3928{\dot{m}}_{CO_{2},lung}-546{\dot{m}}_{N_{2},lung}$$

Linear regression was performed in the data given by Fritts et al. [12] in order to obtain an estimation of work done into the lungs as a function of the minute respiratory ventilation:

(5) $${\dot{W}}_{res}=0.755\exp\left(0.196{\dot{V}}_{E}/Ar_{s}\right)Ar_{s}$$

where the power (*W_res_*) is given in *W* and minute respiratory ventilation (*V_E_*) in L/min.

The air is taken as an ideal gas, and its exergy is equal to the sum of its components, which are modeled as ideal gases, so exergy is a function of specific heat, temperature, partial pressure, and the universal gas constant:

(6) $${\dot{B}}_{a}={\dot{B}}_{O_{2}}+{\dot{B}}_{CO_{2}}+{\dot{B}}_{N_{2}}+{\dot{B}}_{H_{2}O}$$

(7) $${\dot{B}}_{g}={\dot{m}}_{g}\left[c_{p,g}\left(T_{a}-T_{0}-T_{0}\ln\left(\frac{T_{a}}{T_{0}}\right)\right)+R_{g}\;T_{0}\ln\frac{P_{g,a}}{P_{g,0}}\right]$$

where g refers to the gas that is a component of the air. Since the environment is the model reference, the exergy of inhaled air is zero:

(8) $${\dot{B}}_{a,in}=0$$

### 2.1. Methods to Apply the Exergy Analysis to Blood

Blood is a mixture of different substances, for instance, hemoglobin (and other cells), oxygen reacted with hemoglobin, and carbon dioxide reacted with hemoglobin, these gases are dissolved in the blood. Moreover, when there is carbon monoxide in the blood, it is dissolved and reacts with hemoglobin as oxygen does. These methods are adapted from literature or proposed by the authors of this paper and are described in more detail, several mechanisms are based in [13,14,15,16,17].

#### 2.1.1. Exergy of the Mixture of Blood and Ideal Gases

This is a consideration already present in the literature [6,9]. The blood is modeled as a mixture of liquid and gases, and its exergy is given by Equations (9)–(11), where the index g represents each gas. Equation (10) represents the exergy of the liquid phase of the blood, and Equation (11), the exergy of each gas that is dissolved in it:

(9) $${\dot{B}}_{bl}={\dot{B}}_{liq}+{\dot{B}}_{O_{2}}+{\dot{B}}_{CO_{2}}+{\dot{B}}_{CO_{}}$$

(10) $${\dot{B}}_{liq}=\dot{m}\left[c\left(T_{a}-T_{o}-T_{o}\ln\frac{T_{a}}{To}\right)\right]$$

(11) $${\dot{B}}_{g}=\dot{m}\left[c\left(T_{a}-T_{o}-T_{o}\ln\frac{T_{a}}{To}\right)+R_{g}T_{o}\ln\frac{P_{g,s}}{P_{g,o}}\right]$$

#### 2.1.2. Exergy of the Mixture of Blood, Ideal Gases, and Reactions with Hemoglobin

The exergy variation of any reaction is, in fact, the Gibbs free energy change [7,16,17] when the temperature is *T*_0_. Therefore, Equation (12) can be applied to any reaction. This equation indicates that the Gibbs change in a reaction is the opposite of the exergy change:

(12) Δb=−Δg

The method proposed by Albert [17] is used to evaluate the Gibbs free energy variation of the oxygenation process. Equation (13) relates the equilibrium constant to the change of free energy, therefore incorporating into the exergy analysis the term related to the fact that oxygen is not solely transported dissolved in the blood. Moreover, the reaction of hemoglobin is an evolutionary behavior to maintain the concentration difference of the blood and the air within the lungs. In this equation, the term 0 refers to the biological reference for the thermodynamic properties, *R* is the universal gas constant, *T*_0_ is the environmental temperature, and *K*′ is the modified equilibrium constant for the biological reference. The apostrophe in the equilibrium constant refers to constant pH:

(13) $$\Delta_{r}g^{\prime o}=\sum -RT_{0}\ln K^{\prime }$$

Oxygen binds to hemoglobin with increasing ease, a process called cooperativity. There are several models to explain this process. In this work, the model of successive oxygenation steps is taken to explain the oxygenation process.

The change in free energy can also be written regarding Gibbs free energy of formation, as in Equation (14), which is already applied to the oxygenation process:

(14) $$\Delta_{r}G^{\prime o}=\Delta_{f}G^{\prime o}\left(Hb\left(O_{2}\right)\right)-\Delta_{f}G^{\prime o}\left(Hb\right)-\Delta_{f}G^{\prime o}\left(O_{2}\right)$$

the free energy of formation for the unbound hemoglobin, $\Delta_{f}G^{\prime o}\left(Hb\right)$, is taken to be zero [17]. According to Wagman et al. [18], the formation free energy of oxygen for conditions close to the actual human body state, $\Delta_{f}G^{\prime o}\left(O_{2}\right)$, is 16.1 kJ/mol. Equilibrium constants given by Perrella et al. [19] were used because they follow the successive method and provide information for both carbon monoxide and oxygen. These constants are given in Table 1, where it can be concluded that the first reaction demands more energy than the others. With this table, it is possible to assess the Gibbs free energy of the hemoglobin bound with oxygen $\left(HbO_{2}\right)$.

The free energy of formation for each step of oxygenation is given by Equation (15), using the constant values presented in Table 1:

(15) $$\Delta_{f}G^{\prime o}\left(Hb{\left(O_{2}\right)}_{i}\right)=-RT_{0}\ln K_{i}^{\prime }+\Delta_{f}G^{\prime o}\left(Hb{\left(O_{2}\right)}_{i-1}\right)+\Delta_{f}G^{\prime o}\left(O_{2}\right)$$

This equation was applied to all oxygenation steps for oxygen and for carbon monoxide bound with hemoglobin, and the results obtained are shown in Table 2. The term E is the chemical element, CO or O_2_.

To evaluate the exergy variation of the blood, it was considered that hemoglobin is either unbound or fully bound (0 or 4 oxygens). It is important to highlight that the first binding to hemoglobin changes its structure and facilitates the second, third, and fourth reactions, so most of the hemoglobin is in one of these forms. Hence, the exergy change is given by the difference in the number of moles of oxygen present in the arterial to the venous blood, as can be seen in Equation (16), where n represents the number of kmols of a gas in the blood, and Δ*B_E_* the exergy variation for each gas:

(16) $$\Delta B_{E}=\frac{\left(n_{art}-n_{ven}\right)}{4}\left[-\Delta_{f}G^{\prime o}{\left(Hb\left(E\right)\right)}_{4}\right]$$

Carbon dioxide is mostly transported as bicarbonate ions in the blood, according to the reaction represented in Equation (17). It is important to note that carbon dioxide is carried by hemoglobin (10%), but as a simplification, it was not considered here:

(17) $$H^{+}+HCO_{3}^{-}↔CO_{2}+H_{2}O$$

Using the NBS table [20] as a reference for this reaction, it was possible to calculate the Gibbs variation as −44.718 kJ/kmol. The exergy difference in the blood due to carbon dioxide can be represented by Equation (18):

(18) $$\Delta B_{CO_{2}}=(m_{art}-m_{ven})\left(-{\Delta G}_{f}{}_{CO_{2}}\right)$$

Eventually, it was possible to assess the exergy variation between the venous blood and the arterial blood according to Equation (19):

(19) $$\Delta {\dot{B}}_{Hb}=\Delta {\dot{B}}_{O_{2}}+\Delta {\dot{B}}_{CO_{}}+\Delta {\dot{B}}_{CO_{2}}$$

Eventually, it was possible to obtain the destroyed exergy rate for the lungs according to Equation (20):

(20) $${\dot{B}}_{d,lung}={\dot{B}}_{M,lung}+{\dot{B}}_{bl,ven}+{\dot{B}}_{a,in}+{\dot{W}}_{resp}-{\dot{B}}_{bl,art}-{\dot{B}}_{a,ex}-{\dot{Q}}_{M,lung}\left(1-\frac{T_{0}}{T_{\mathrm{lung}}}\right)-\Delta {\dot{B}}_{Hb}$$

### 2.2. Second Law of Thermodynamics of the Lung

In this analysis, only the gas diffusion between two compartments, lung alveoli and arterial blood, was considered. Therefore, it was possible to pinpoint the irreversibilities associated with the differences of chemical potential. For carbon dioxide, the entropy variation is evaluated according to Equation (21):

(21) $$\Delta {\dot{S}}_{CO_{2}}=-\left({\dot{m}}_{co_{2}v}-{\dot{m}}_{co_{2}a}\right)R_{CO_{2}}\ln\frac{P_{co_{2},ex}}{P_{co_{2},art}}$$

This gas concentration is higher in the venous blood, and it diffuses from the blood to the alveoli. For both O_2_ and CO, the concentration is higher in the arterial blood, and it diffuses from the lungs to the blood, hence the entropy variation was calculated according to Equations (22) and (23):

(22) $$\Delta {\dot{S}}_{CO_{}}=-\left({\dot{m}}_{coa}-{\dot{m}}_{cov}\right)R_{CO_{}}\ln\frac{P_{co},art}{P_{co,ex}}$$

(23) $$\Delta {\dot{S}}_{O_{2}}=-\left({\dot{m}}_{o_{2}a}-{\dot{m}}_{o_{2}v}\right)R_{O_{2}}\ln\frac{P_{o_{2,}art}}{P_{o_{2},ex}}$$

The total entropy production rate is given by Equation (24) and the destroyed exergy by Equation (25):

(24) $$\dot{\sigma }=\Delta {\dot{S}}_{CO_{2}}+\Delta {\dot{S}}_{CO_{}}+\Delta {\dot{S}}_{O_{2}}$$

(25) $${\dot{B}}_{d}=T_{o}\dot{\sigma }$$

### 2.3. Simulations

The exergy balance was applied to different scenarios, resulting in exergy destroyed rates as a function of altitude, acclimatization time (level of hemoglobin in the blood), and the concentration of carbon monoxide in the air. Four altitudes were considered: 0, 1500, 3000, and 4500 m, associated with different pressures, as shown in Table 3.

The concentration of hemoglobin in the blood as a function of time and altitude is given in Table 4 [9]. It is important to highlight that the amount of hemoglobin increases with altitude for a given altitude higher than sea level. This is a physiological response to the decreased amount of oxygen in the atmosphere. Ventilation rate is another acclimatization mechanism [13,14]. It increases sharply in the first minutes and then plunges to a value that is higher than the average value at sea level, as indicated in Table 4. This rate increases linearly until the 28th day of exposure, when it becomes constant. It may drop to an intermediate value if the exposure to high altitude lasts for a long period (years). It is more significant for altitudes higher than 1500 m. Therefore, it was admitted to be constant and equal at 0 and 1500 m. There are data available for the highest altitude [14], but none were found for the intermediate one. A linear interpolation was made to estimate the values for 3000 m.

For each concentration, 11 simulations were carried out, varying the quantity of inhaled carbon monoxide from 0 to 100 ppm. The upper limit is high, equal to 300% of the value considered critical in the environmental standards. The information required to calculate these efficiencies was obtained from the Albuquerque et al. [11] model. The input data are the physiological parameters, the inhaled air composition, and the environmental pressure.

## 3. Results and Discussion

The simulation results were used to calculate the destroyed exergy rate for each of the studied cases, that is, altitude and different concentrations of hemoglobin and carbon monoxide. As mentioned above, three approaches to exergy analysis of the lung were taken. The first is a method already described in the literature [9]: For each case it is possible to define exergy efficiency as the ratio between exergy output and exergy input. The second and third models were proposed by this author. For these methods, exergy efficiency was not defined, in the first case because just the exergy difference between arterial blood and venous blood was calculated, making it impossible to define exergy as a rate between outputs and inputs, and in the third case because the method used was a second law balance, which gives an entropy production rate, which is related to exergy by Equation (25), but does not allow defining meaningful efficiency.

### 3.1. Considering Gases in the Blood as Ideal Gases

While for the phenomenological model, the reaction of hemoglobin with carbon monoxide and oxygen was considered, the initial literature [6,9] did not take into account the reaction of hemoglobin with these gases in the exergy analysis, for simplification. The exergy efficiency was only evaluated for the present model based on [6,9]. The definition was the ratio of flow rates that leave the control volume to those who enter it. The reason for demonstrating only this item is its applicability to the model considering the blood and gases as a mixture of ideal gases as proposed in Equation (26):

(26) $$\eta =\frac{{\dot{B}}_{sga}+{\dot{B}}_{a,exp}+{\dot{Q}}_{M,ling}\left(1-\frac{T_{a}}{T_{c}}\right)}{{\dot{M}}_{lung}+{\dot{W}}_{resp}+{\dot{B}}_{sgv}}$$

**Figure 4:**
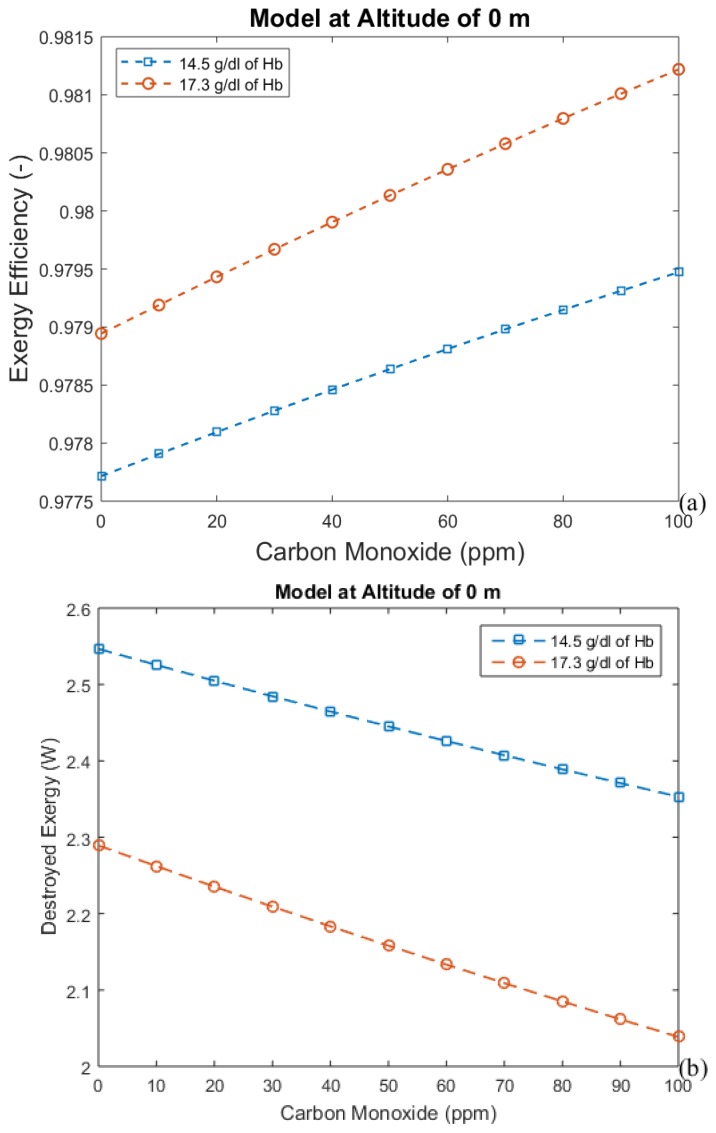
(**a**) Exergy efficiency and (**b**) destroyed exergy as functions of carbon monoxide and hemoglobin (Hb) concentration at sea level.

**Figure 5:**
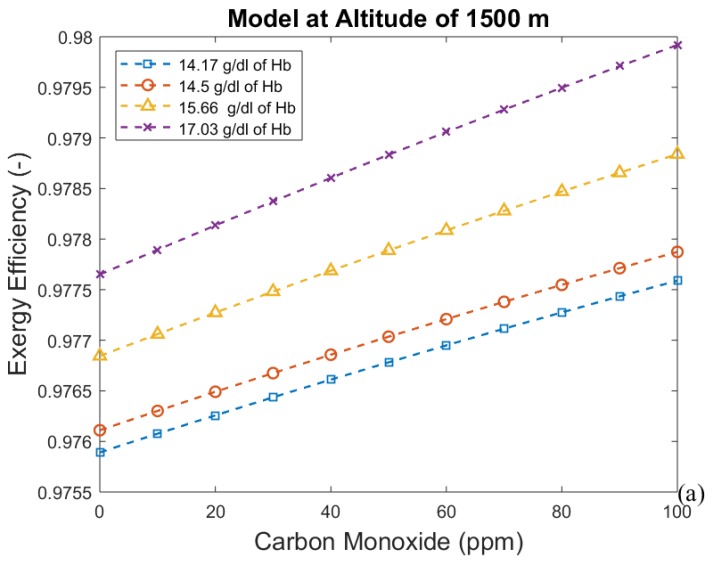

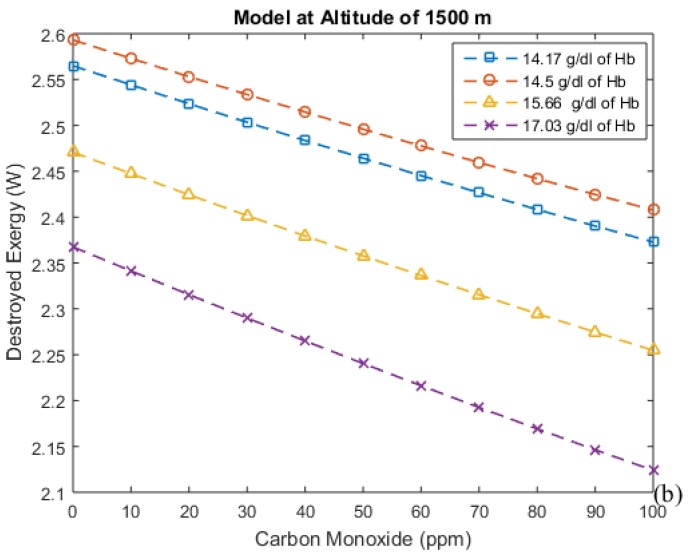
(**a**) Exergy efficiency and (**b**) destroyed exergy as functions of carbon monoxide and hemoglobin concentration at 1500 m.

**Figure 6:**
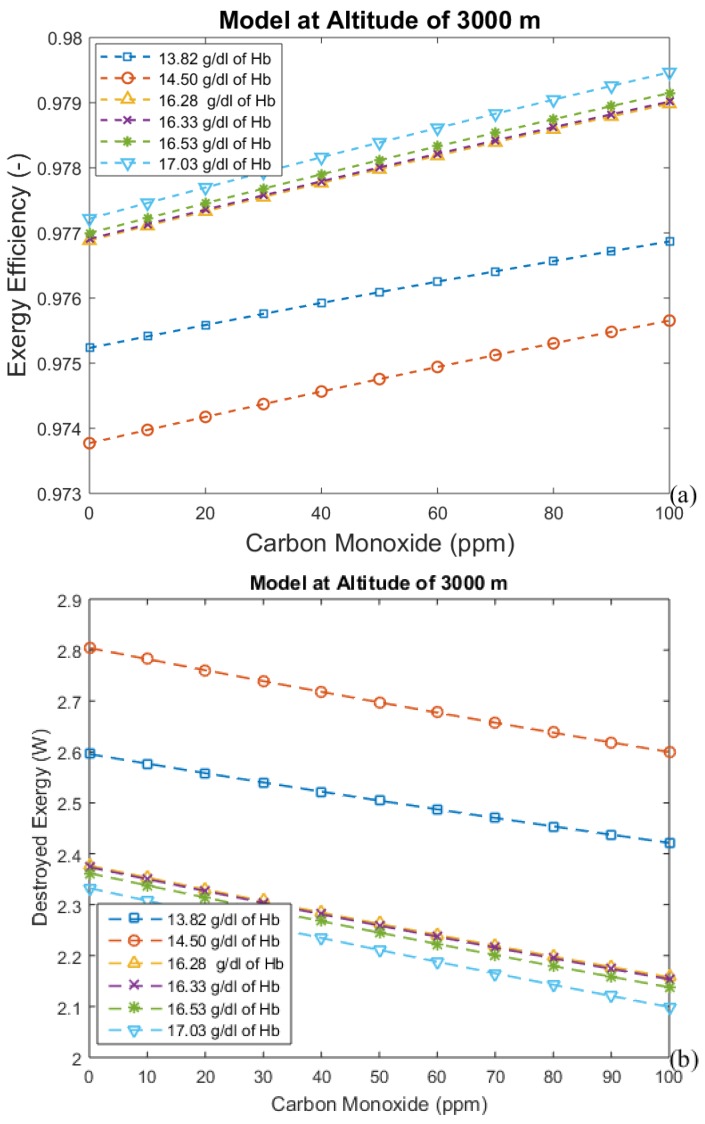
(**a**) Exergy efficiency and (**b**) destroyed exergy as functions of carbon monoxide and hemoglobin concentration at 3000 m.

**Figure 7:**
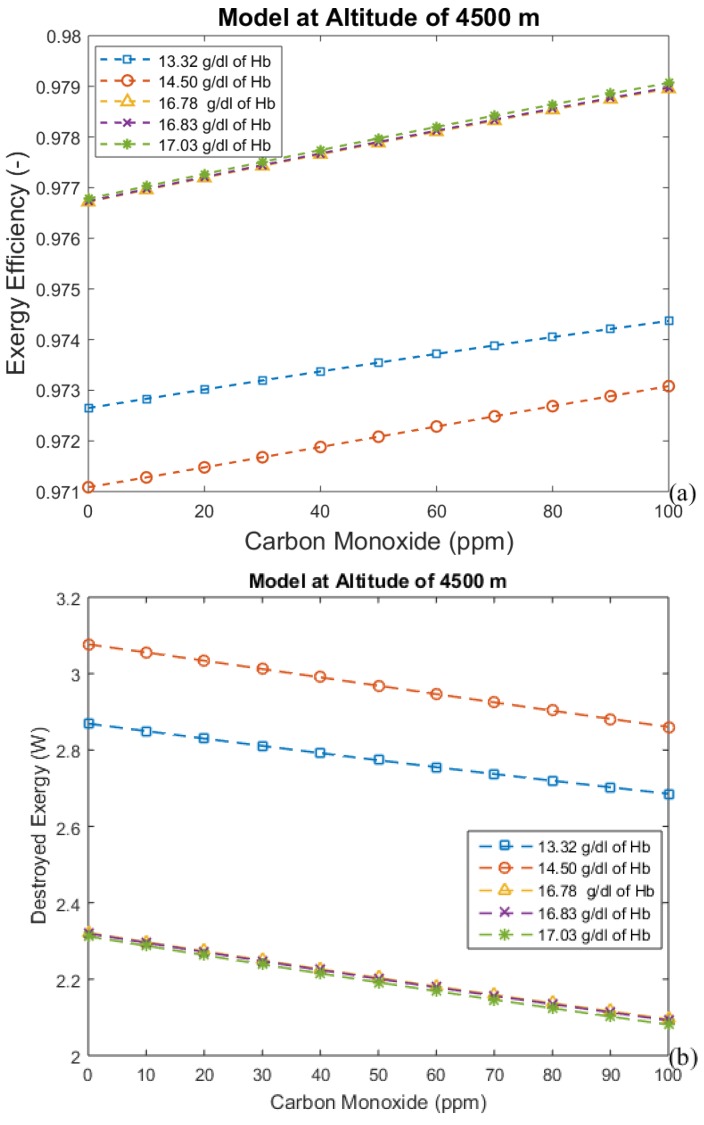
(**a**) Exergy efficiency and (**b**) destroyed exergy as functions of carbon monoxide and hemoglobin concentration at 4500 m.

The hemoglobin concentration changes with the period of acclimatization to different altitudes, increasing with time. These results are shown in Figure 4, Figure 5, Figure 6 and Figure 7, where time is represented as the acclimatization period, and an increase in hemoglobin concentration can be seen. Each graph considers the model at a certain altitude with different amounts of hemoglobin. More hemoglobin potentially means more time spent at high altitudes. From these graphs we can conclude that for all altitudes and hemoglobin concentrations, efficiency increases when carbon monoxide intoxication becomes more severe, whereas the exergy destroyed in the lungs decreases. This is an unexpected result, since CO is associated with suffocation.

Figure 4, Figure 5, Figure 6 and Figure 7 indicate the exergy destroyed by the respiratory system (and exergy efficiency), *B_d_* (in Watts), as a function of carbon monoxide at different hemoglobin concentrations. The exergy destruction rate decreases at higher altitudes. Carbon monoxide has been shown to be harmful to exergy behavior at all altitudes and hemoglobin concentrations. Exergy destruction increases with higher concentrations of hemoglobin and carbon monoxide. The bind that is created between carbon monoxide and hemoglobin reduces oxygen absorption. Therefore, a larger amount of hemoglobin can be related to an increase in the total amount of carbon monoxide in the blood. This suggests that people who spend time in areas with significant amounts of carbon monoxide may have higher concentrations of hemoglobin to compensate for the inefficiency associated with the low concentration of oxygen in the blood.

### 3.2. Considering the Effect of Hemoglobin Reaction with Inspired Gases

**Figure 8:**
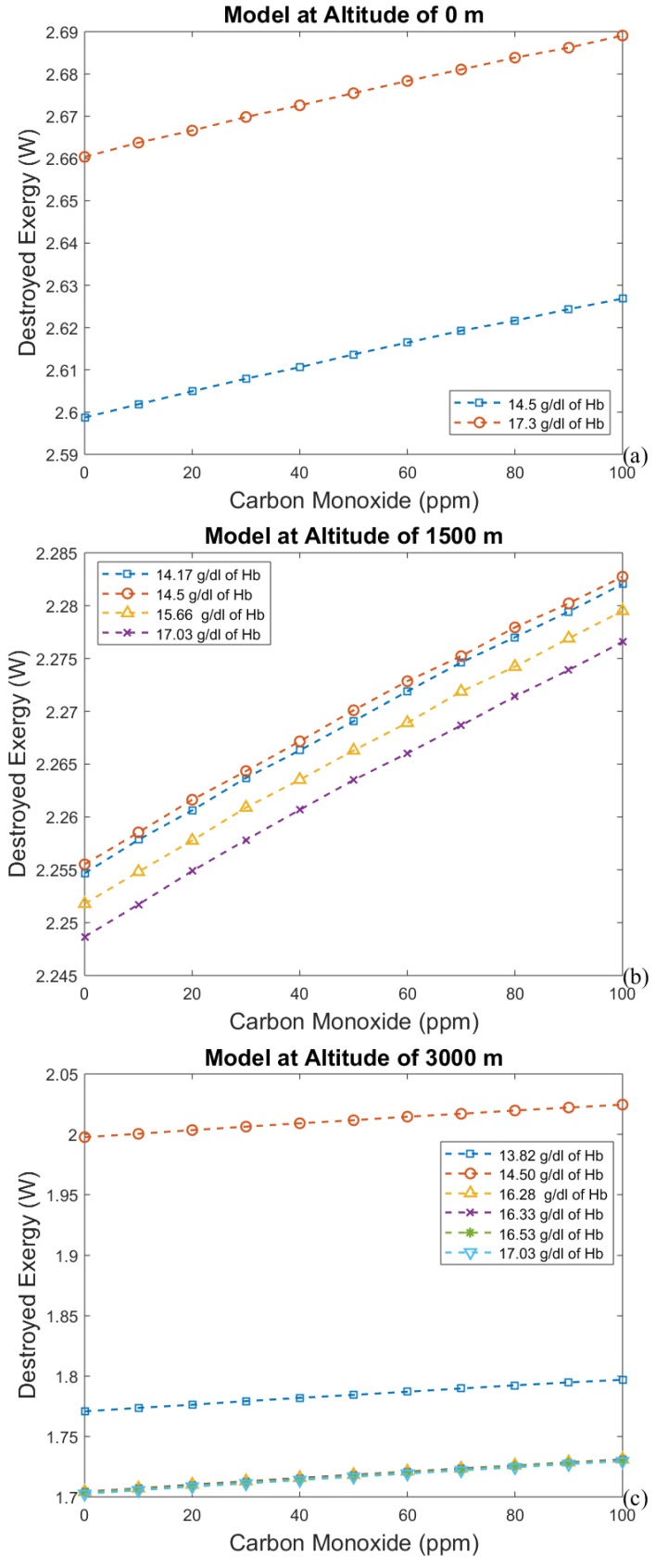

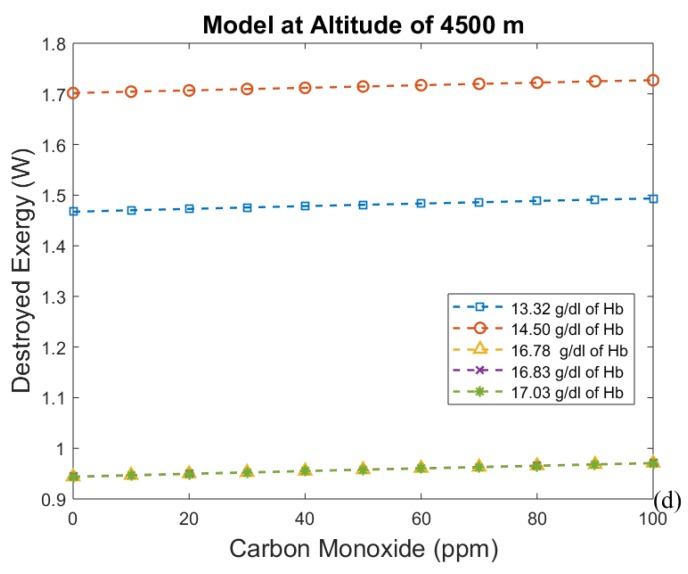
Destroyed exergy as a function of carbon monoxide and hemoglobin concentration at (**a**) 0 m, (**b**) 1500 m, (**c**) 3000 m, and (**d**) 4500 m.

Figure 8 indicates the effect of carbon monoxide and altitude on the exergy behavior of the human body at different altitudes and acclimatization times. At an altitude of 0 m with 17.03 g/dL of hemoglobin, there is a small difference in the destroyed exergy of the lungs. It is important to highlight that this indicates that the person has returned from 4500 m to 0 m. The consequence is a higher amount of hemoglobin in the blood. Another important issue is regarding the amount of destroyed exergy in the lungs as a function of carbon monoxide intoxication. It is important to note that hemoglobin is in part responsible for the decreases in the change of destroyed exergy with the intoxication level, and in the absolute value of destroyed exergy in the lungs. Moreover, from an evolutionary point of view, the presence of hemoglobin is related to maintaining the gradient pressure between the air in the lungs and the blood; therefore, higher amounts of hemoglobin would facilitate this phenomenon. Nevertheless, it is important to point out that the reaction with hemoglobin is necessarily more reversible than the simple gas transport mechanism, which can be identified in Figure 8a–d, as a reduction in the overall destroyed exergy of the lungs.

### 3.3. Irreversibilities Associated with Mass Transfer Rate

**Figure 9:**
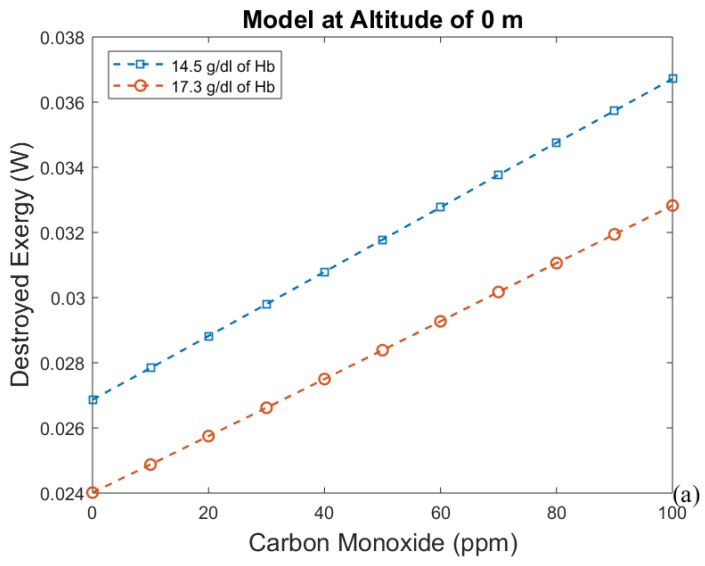

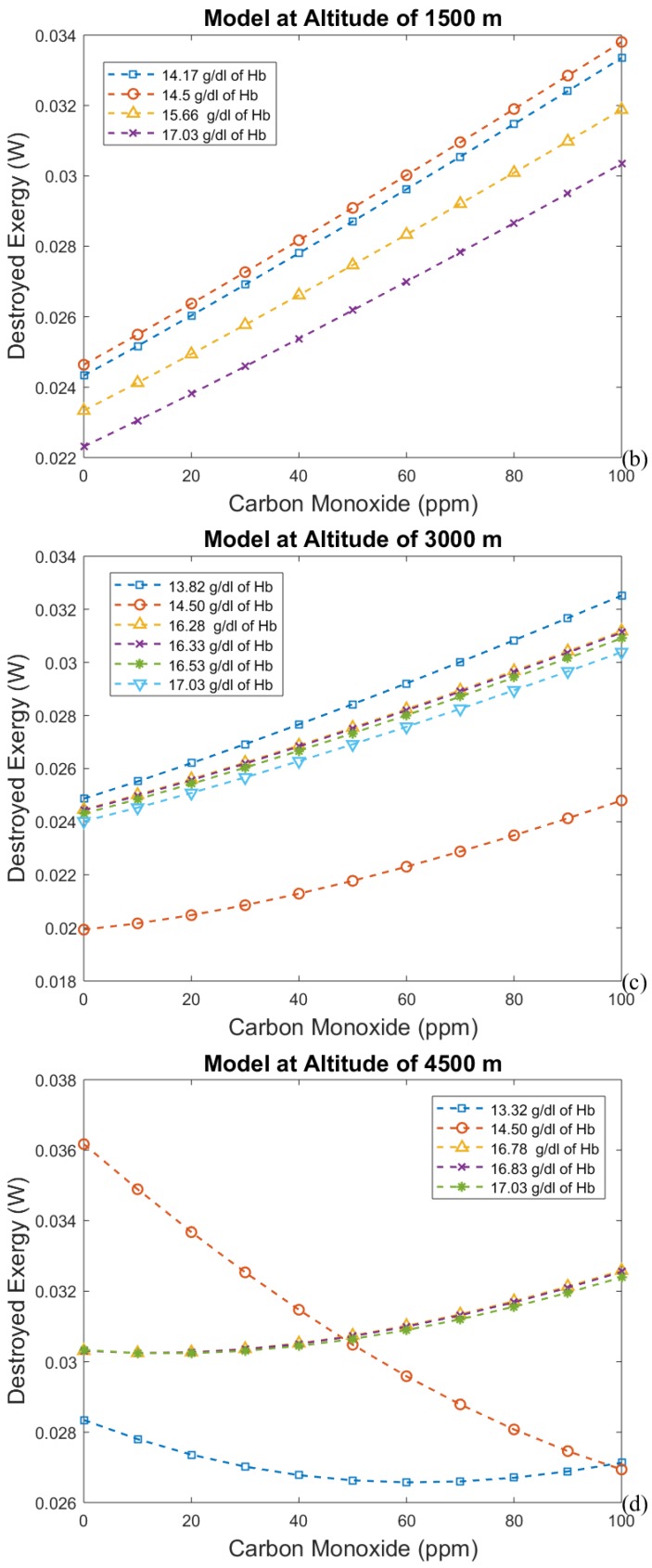
Destroyed exergy as function of carbon monoxide and hemoglobin concentration at (**a**) 0 m, (**b**) 1500 m, (**c**) 3000 m, and (**d**) 4500 m.

A comparison of the model, considering the amount of hemoglobin in the exergy method and the gases dissolved in the blood as ideal gases, indicates that it is necessary to study only the irreversibilities associated with the transport phenomena, bearing in mind that the phenomenological model proposed by [11] takes into account these parameters, although the first methods to perform exergy analysis only considered that all the gases are dissolved in the blood. Figure 9a,b indicates that an increase in the amount of hemoglobin is directly related to a decrease in the total destroyed exergy associated with a difference of chemical potential of the gases in the air and the blood within the lungs. One interesting result is the increase of destroyed exergy with the increase of CO concentration in the air. A possible explanation can be based in the fact that there is an increase in mass transfer to the lungs. At an altitude of 4500 m, there is a modification in the trend, probably because the physiological model used may not be appropriate for such altitudes, and this method is more sensitive to pressure differences. Another reason is that modifications in physiology are more severe at this level, and therefore what is seen in Figure 9d is a combination of all physiologic responses (which is more plausible than that the model is not validated for these heights).

## 4. Conclusions

In this work, one method for the exergetic analysis of the human body was adapted and applied in order to evaluate different concentrations of carbon monoxide and its hazards associated with altitude. This method was applied to the model proposed by Albuquerque et al. [11], which was used to simulate the effects of carbon monoxide intoxication at different altitudes on exergy efficiency and destroyed exergy of the respiratory system. Later, three approaches were taken to the exergy of the blood. In the first, it was considered as an ideal mixture of liquid and ideal gases. The second considered the effects of carbon monoxide and oxygen association on hemoglobin and carbon dioxide dissociation. The third was a second law approach to the mass transfer that occurs in the lungs.

- The first approach leads to an increase in efficiency (and a decrease in exergy destruction rate) when CO poisoning happens. Acclimatization increases efficiency and altitude decreases it.
- For the second model, a significant reduction in the effects of CO poisoning can be seen, though it is responsible for a slight increase in the exergy destruction rate. In this model, as in the previous one, acclimatization (increased hemoglobin concentration and ventilation) has a positive effect on the respiratory system, reducing the exergy destruction rate.
- The third analysis gives results that agree with the results of the second one. So, it is possible to conclude that using these two new approaches will lead to a significant discovery in the analysis of the human body.

## Figures and Tables

**Table 1:** Equilibrium constants for successive reactions of gases (carbon monoxide and oxygen) with hemoglobin.

Equilibrium Constant	CO	O_2_
$K_{41}^{\prime }$	4.82 × 10^5^	1.96 × 10^6^
$K_{42}^{\prime }$	1.61 × 10^6^	1.25 × 10^6^
$K_{43}^{\prime }$	2.50 × 10^8^	2.68 × 10^7^
$K_{44}^{\prime }$	4.64 × 10^8^	1.52 × 10^9^

**Table 2:** Gibbs free energy variation in successive reactions of gases (carbon monoxide and oxygen) with hemoglobin.

Gibbs Free Energy Variation	CO	O_2_
$\Delta_{f}G^{\prime o}\left(Hb\left(E\right)\right)$	−152.00	−19.44
$\Delta_{f}G^{\prime o}{\left(Hb\left(E\right)\right)}_{2}$	−306.95	−37.78
$\Delta_{f}G^{\prime o}{\left(Hb\left(E\right)\right)}_{3}$	−474.23	−63.63
$\Delta_{f}G^{\prime o}{\left(Hb\left(E\right)\right)}_{4}$	−643.12	−99.38

**Table 3:** Atmospheric pressure as a function of altitude.

Altitude (m)	Atmospheric Pressure (kPa)
0	101.3
1500	84.5
3000	70.1
4500	57.7

**Table 4:** Concentration of hemoglobin in the blood as function of time and altitude. Based on and adapted from [9,13,14].

Altitude (m)	Time (days)	Hemoglobin Concentration (g/dL)	Alveolar Ventilation (mL/min)
0	0	14.5 (reference value)	4212.0
0	2	14.5	4212.0
0	20	14.5	4212.0
0	60	14.5	4212.0
0	90	14.5	4212.0
0	0	17.03	4212.0
1500	0	14.5	5300.0
1500	2	14.17	5300.0
1500	20	15.66	5300.0
1500	60	15.66	5300.0
1500	90	15.66	5300.0
1500	0	17.03	5300.0
3000	0	14.5	5850.0
3000	2	13.82	6900.0
3000	20	16.28	7162.5
3000	60	16.33	7162.5
3000	90	16.53	7162.5
3000	0	17.03	7162.5
4500	0	14.5	6525.0
4500	2	13.32	7575.0
4500	20	16.78	9675.0
4500	60	16.83	9675.0
4500	90	17.03	9675.0

## Nomenclature

A	altitude, m
Ar	area, m^2^
b	specific exergy, J/kg
**B**	exergy, J
$\dot{B}$	exergy rate and flow rate, W
c	specific heat capacity, J/(kg K)
G	Gibbs free energy, J
g	specific Gibbs free energy, J/kg or J/kmol
H	enthalpy flow rate, W
K	equilibrium constant
M	metabolism, W
M	mass flow rate, kg/s
$\dot{Q}$	heat transfer rate, W
P	pressure, Pa
R_i_	gas constant of the molecule i, J/(kgK)
S	entropy, J/K
s	specific entropy J/(kgK)
T	temperature, °C or K
V	respiratory ventilation, L/min
T	time, s
$\dot{W}$	performed power, W
Greek symbols	
Δ	variation
σ	entropy production rate, W/K
η	efficiency
Subscripts and superscripts	
0	reference
a	air
art	arterial
body	body
bl	blood
CO	carbon monoxide
CO_2_	carbon dioxide
D	destroyed
ex	expired
f	formation
g	gas
H_2_O	water
in	inflow
liq	liquid
lung	lung
M	metabolic
N_2_	nitrogen
O_2_	oxygen
out	outflow
resp	respiratory
ven	venous
